# Supplementary material for: Genetic variance in the murine defensin locus modulates glucose homeostasis
Source: EMBO J. 2025 Sep 9;44(20):5694–711. doi: 10.1038/s44318-025-00555-5 (PMC12528722; doi:10.1038/s44318-025-00555-5)
Supplement: Supplementary file 1 — Appendix [file 44318_2025_555_MOESM1_ESM.pdf]

Appendix for Genetic variance in the murine defensin locus modulates glucose homeostasis

Table of Contents:

Page 2: ....Appendix Figure S1

Page: 4..Appendix Figure S2

Page: 6..Appendix Figure S3

Page: 8..Appendix Figure S4

Page: 10..Appendix Figure S5

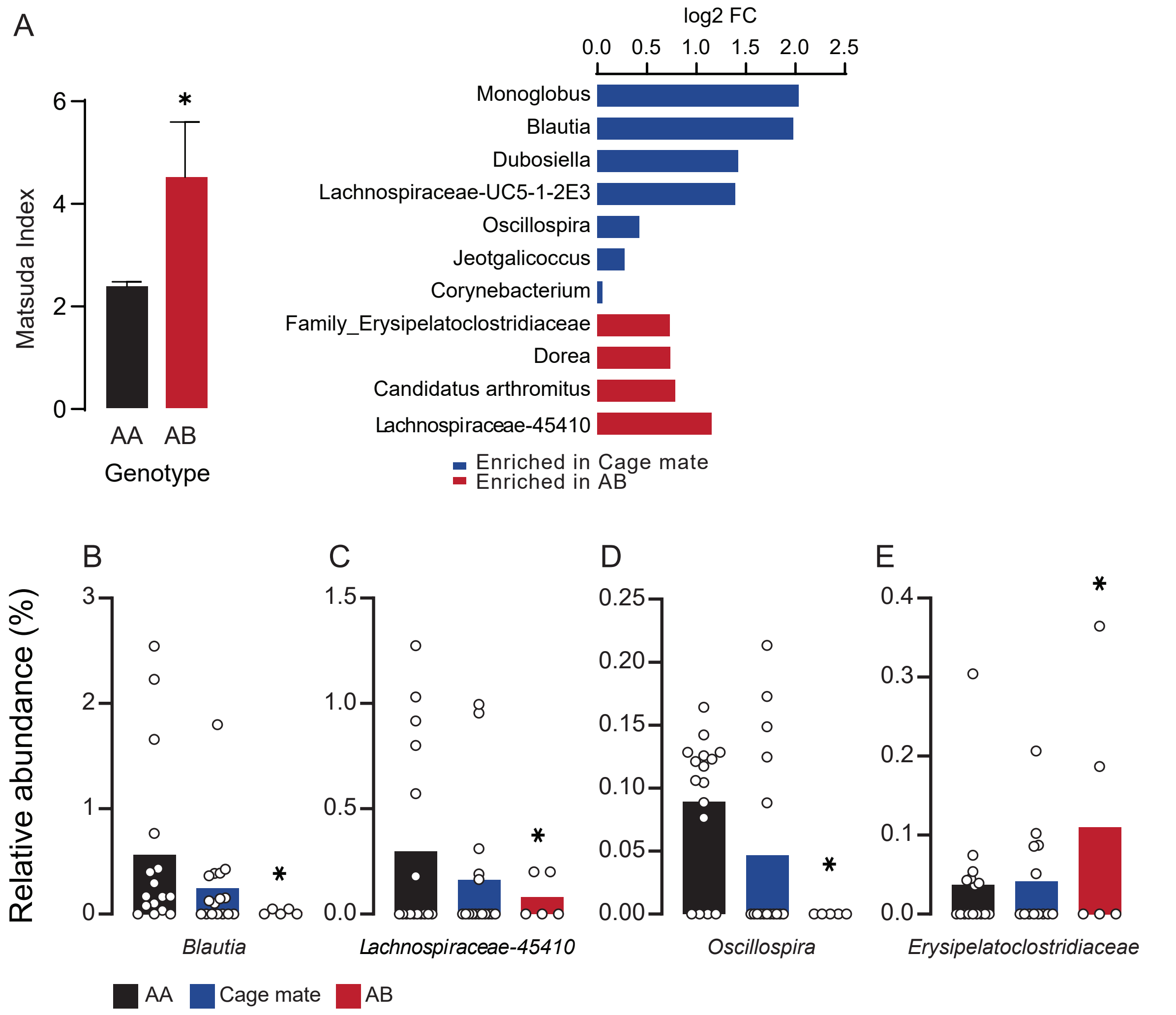
A
Matsuda Index
AA
AB
Genotype
log2 FC
Monoglobus
Blautia
Dubosiella
Lachnospiraceae-UC5-1-2E3
Oscillospira
Jeotgalicoccus
Corynebacterium
Family_Erysipelatoclostridiaceae
Dorea
Candidatus arthromitus
Lachnospiraceae-45410
Enriched in Cage mate
Enriched in AB
B
C
D
E
Relative abundance (%)
Blautia
Lachnospiraceae-45410
Oscillospira
Erysipelatoclostridiaceae
AA
Cage mate
AB

Appendix Figure S1: Microbiome differences between AB and their cage mates. A) Difference (log2 FC) in relative abundance of microbes identified by Analysis of Compositions of Microbiomes with Bias Correction (ANCOM-BC) between C57BL6/J and A/J mice fed WD + Defa26 for eight weeks. **B)** Relative abundance of Blautia sp. **C)** Lachnospiraceae-45410 sp. **D)** Oscillospira sp. and **E)** Erysipelatoclostridiaceae sp. in AA, Cage mate, and AB mice. Data are mean, error bars represent SD of difference between groups, biological replicates shown as individual data points. * $P < 0.05$ compared to cage mates.

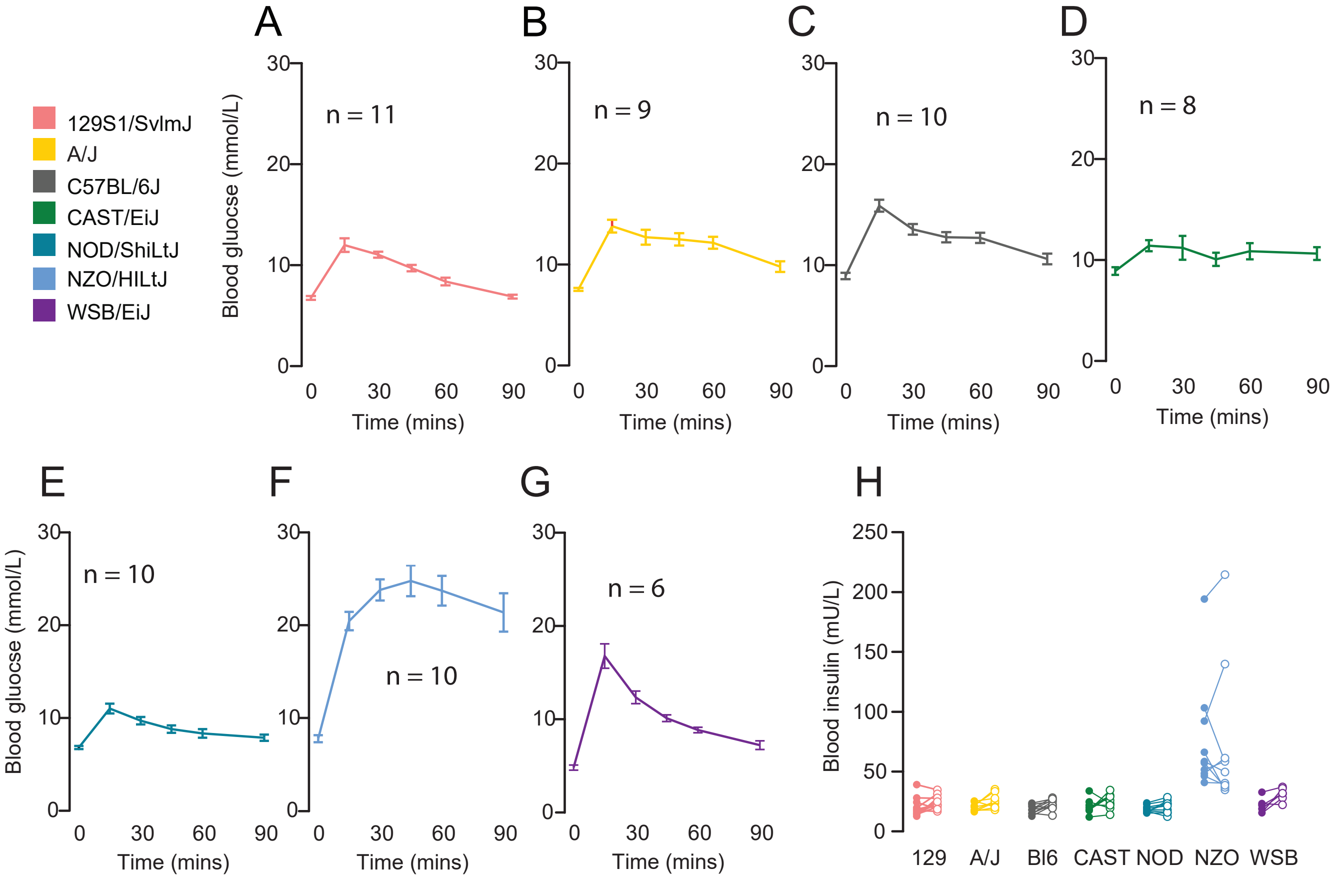
129S1/SvlmJ
A/J
C57BL/6J
CAST/EiJ
NOD/ShiLtJ
NZO/HILtJ
WSB/EiJ
A
B
C
D
E
F
G
H
n = 11
n = 9
n = 10
n = 8
n = 10
n = 10
n = 6
Blood gluocse (mmol/L)
Time (mins)
Blood insulin (mU/L)
129
A/J
Bl6
CAST
NOD
NZO
WSB

Appendix Figure S2: Glucose tolerance and blood insulin concentrations in Diversity Outbred founder strains. Blood glucose concentrations during a glucose tolerance test in **A)** 129S1/SvImJ, **B)** A/J, **C)** C57BL/6J, **D)** CAST/EiJ, **E)** NOD/ShiLtJ, **F)** NZO/HILtJ, **G)** WSB/EiJ. **H)** Insulin concentration in Diversity Outbred founder strains during a glucose tolerance test. Data are mean with biological replicates are shown as individual data points or noted in figure.

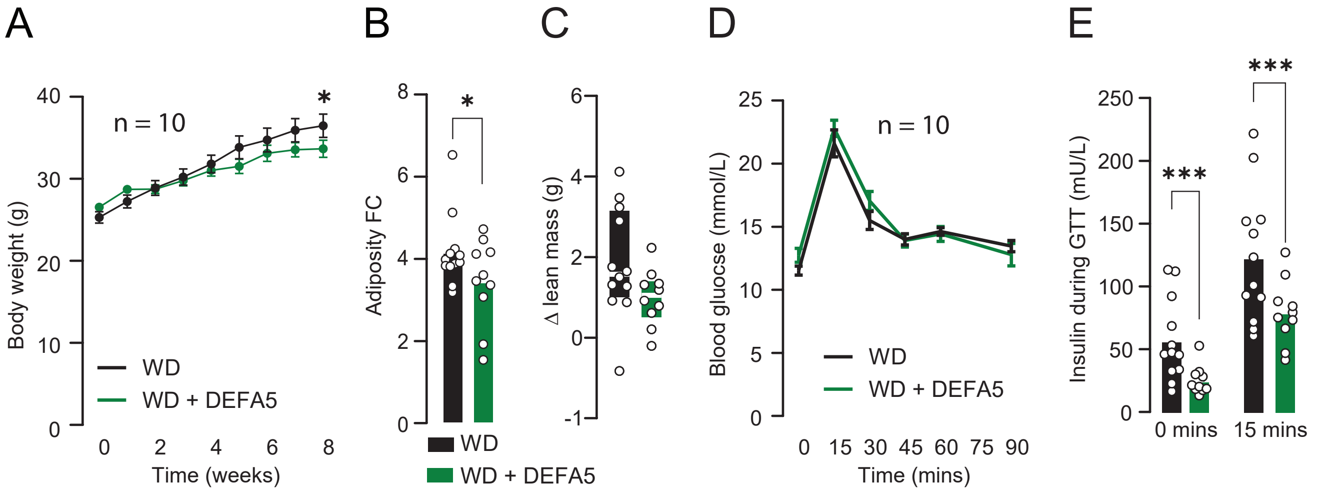

A
n = 10
Body weight (g)
Time (weeks)
WD
WD + DEFA5
B
Adiposity FC
C
Δ lean mass (g)
WD
WD + DEFA5
D
n = 10
Blood gluocse (mmol/L)
Time (mins)
WD
WD + DEFA5
E
Insulin during GTT (mU/L)
0 mins
15 mins

Appendix Figure S3: Effect of alpha-defensin 5 supplementation on insulin sensitivity and body composition in C57BL/6J mice. A) Body weight of mice fed either WD or WD+DEFA5. **B)** Fold-change in adiposity in mice fed either WD or WD+DEFA5. **C)** Change in lean mass in mice fed either WD or WD +DEFA5. **D)** Blood and **E)** Insulin concentrations in mice fed either WD or WD +DEFA5 during a glucose tolerance test. Data are mean with biological replicates are shown as individual data points or in figure. *** $P < 0.001$, * $P < 0.05$. Metabolic phenotypes were compared by two-way RM ANOVA with post-hoc Student's t-test (A, D), two-way ANOVA with Tukey's LSD (E), or Student's t-test (B, C).

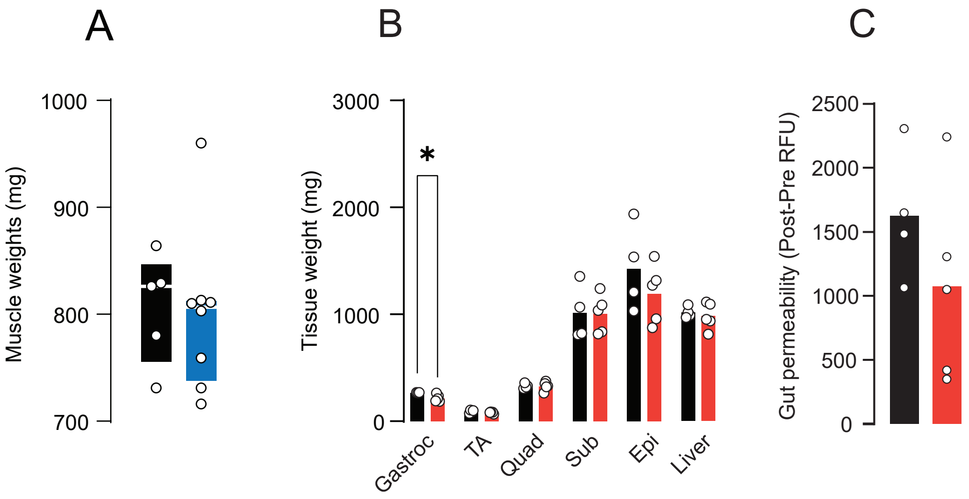

A
B
C
Muscle weights (mg)
1000
900
800
700
Tissue weight (mg)
3000
2000
1000
0
*
Gastroc
TA
Quad
Sub
Epi
Liver
Gut permeability (Post-Pre RFU)
2500
2000
1500
1000
500
0

Appendix Figure S4: Effect of alpha-defensin 29 supplementation on C57BL6/J and A/J tissue mass and gut integrity. A) Combined mass of gastrocnemius, tibialis anterior and quadriceps muscles from C57BL6/J mice after WD or WD + Defa26 feeding for 8 weeks. **B)** Tissue weights of A/J mice fed either a WD or WD + Defa26 for eight weeks. **C)** Relative gut permeability (post – pre FITC fluorescence) of A/J mice fed either a WD or WD + Defa26 for eight weeks. Data are mean with biological replicates shown as individual data points. * P < 0.05 denotes statistical significance from WD control. Tissue weights were compared by Student’s t-test (A) or two-way ANOVA with Tukey's LSD (B). Gut permeability was compared by Student’s t-test (C).

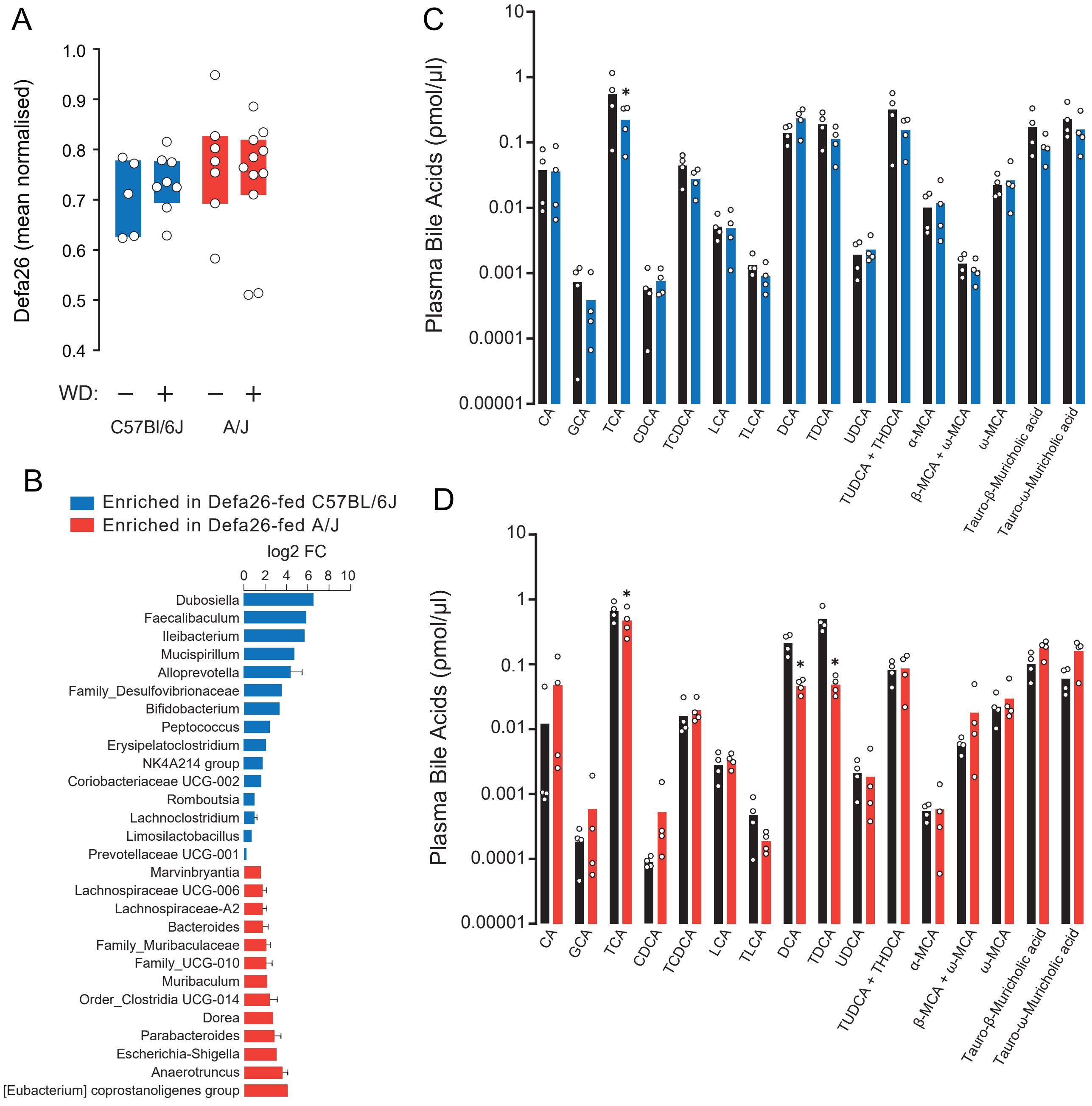

A
Defa26 (mean normalised)
1.0
0.9
0.8
0.7
0.6
0.5
0.4
WD: − + − +
C57Bl/6J
A/J
B
Enriched in Defa26-fed C57BL/6J
Enriched in Defa26-fed A/J
log2 FC
0 2 4 6 8 10
Dubosiella
Faecalibaculum
Ileibacterium
Mucispirillum
Alloprevotella
Family_Desulfovibrionaceae
Bifidobacterium
Peptococcus
Erysipelatoclostridium
NK4A214 group
Coriobacteriaceae UCG-002
Romboutsia
Lachnoclostridium
Limosilactobacillus
Prevotellaceae UCG-001
Marvinbryantia
Lachnospiraceae UCG-006
Lachnospiraceae-A2
Bacteroides
Family_Muribaculaceae
Family_UCG-010
Muribaculum
Order_Clostridia UCG-014
Dorea
Parabacteroides
Escherichia-Shigella
Anaerotruncus
[Eubacterium] coprostanoligenes group
C
Plasma Bile Acids (pmol/µl)
10
1
0.1
0.01
0.001
0.0001
0.00001
CA
GCA
TCA
CDCA
TCDCA
LCA
TLCA
DCA
TDCA
UDCA
TUDCA + THDCA
α-MCA
β-MCA + ω-MCA
ω-MCA
Tauro-β-Muricholic acid
Tauro-ω-Muricholic acid
D
Plasma Bile Acids (pmol/µl)
10
1
0.1
0.01
0.001
0.0001
0.00001
CA
GCA
TCA
CDCA
TCDCA
LCA
TLCA
DCA
TDCA
UDCA
TUDCA + THDCA
α-MCA
β-MCA + ω-MCA
ω-MCA
Tauro-β-Muricholic acid
Tauro-ω-Muricholic acid

Appendix Figure S5: Comparison of C57BL6/J and A/J alpha defensin 26 supplementation responses. A) Endogenous alpha-defensin 26 expression in chow and WD fed C57BL6/J and A/J mice. **B)** Difference (log2 FC) in relative abundance of microbes identified by Analysis of Compositions of Microbiomes with Bias Correction (ANCOM-BC) between C57BL6/J and A/J mice fed WD + Defa26 for eight weeks. **C)** Plasma bile acid concentrations from C57BL/6J mice fed either a WD or WD + Defa26 for eight weeks. **D)** Plasma bile acid concentrations from A/J mice fed either a WD or WD + Defa26 for eight weeks. Data are mean with biological replicates are shown as individual data points or in figure. For differentially abundant microbes, error bars represent SD of difference between groups. * $P < 0.05$ denotes a significant difference from WD fed mice. Alpha-defensin 26 expression and bile acids were compared by two-way ANOVA with Tukey's LSD.
